# Patient perspectives and preferences on cerclage and preterm birth: a focus group study

**DOI:** 10.1007/s11136-024-03637-9

**Published:** 2024-06-18

**Authors:** Nour Abdulrahman, Nicole B. Burger, Susan van den Broek, Eugenie M. Kaaijk, Martijn A. Oudijk, Marjon A. de Boer, Judith A. F. Huirne

**Affiliations:** 1 Department of Obstetrics and Gynaecology, Amsterdam University Medical Center, Vrije Universiteit Amsterdam, Boelelaan 1117, Amsterdam, The Netherlands; 2 Amsterdam Reproduction and Development Research Institute, Meibergdreef 9, Amsterdam, The Netherlands; 3 Department of Obstetrics and Gynaecology, OLVG, Oosterpark 9, 1091 AC Amsterdam, The Netherlands

**Keywords:** Qualitative research, Focus groups, Cervical cerclage, Preterm birth, Miscarriage

## Abstract

**Aim:**

This qualitative focus group study aims to asses cerclage-related symptoms, the impact of a cerclage on daily functioning and patient perspectives of their healthcare experience. This study extends beyond the current focus on surgical and obstetric outcomes of a cerclage, thereby contributing to a more comprehensive understanding of the challenges faced by individuals in the context of extreme preterm birth and fetal loss and the impact of a cerclage on multiple facets in life.

**Methods:**

Participants were recruited from the Amsterdam University Medical Center, Amsterdam, the Netherlands or via the website of a Dutch patient organization for (extreme) preterm birth. Eligible participants were ≥ 18 years old with a previous vaginal and/or abdominal cerclage with a subsequent delivery at ≥ 34 weeks of gestation with neonatal survival. Two focus group discussions (FGD) were performed. A predefined format was used, which was identical for both the vaginal and abdominal cerclage group. The International Classification of Functioning, Disability and Health (ICF-DH) was used to provide structure. Outcomes were a broad range of participants reported perspectives on physical, emotional, and social-related quality of life.

**Results:**

In the Vaginal Cerclage Group (VCG) and Abdominal Cerclage Group (ACG), respectively, 11 and 8 participants were included. Fear for a subsequent pregnancy loss was the most limiting factor to perform daily activities during pregnancy in all participants with a cerclage. Fear to conceive again because of prior second-trimester fetal loss was experienced by 27% in the VCG and 13% in the ACG. The majority of participants experienced a reduction in anxiety after placement of their cerclage (VCG = 64%, ACG = 75%). Decreased mobility/bedrest (VCG = 100%, ACG = 75%) and blood loss (VCG = 55%, ACG = 13%) were frequently mentioned complaints during pregnancy with cerclage. Other aspects mentioned in both groups were social isolation, the lack of societal participation, and the perceived need to quit work and sports. All participants in the abdominal cerclage group reported a lack of comprehensible and unambiguous information about obstetric management and expectations during pregnancy in secondary care hospitals. Clear communication between secondary and tertiary care hospitals about obstetric management following an abdominal cerclage, for example, about the need for cervical length measurements by ultrasound, the need for bedrest or advice concerning sexual activity was missing (63%). Psychologic support was desired in half of all participants, but was not offered to them.

**Conclusions:**

The fear of a subsequent pregnancy loss was reported as the most limiting factor in daily life by all participants. Cerclage placement resulted in the reduction of anxiety. Participants mentioned a significant impact of bedrest and activity restriction during pregnancy with cerclage on social participation and daily activities. Unfortunately, no high level evidence is available on this matter. Patients might even benefit from appropriate levels of physical activity throughout their pregnancy to promote their overall well-being. More evidence is needed to determine the optimal level of physical activity. There is a need for clear and unambiguous patient information about obstetric management.

**Supplementary Information:**

The online version contains supplementary material available at 10.1007/s11136-024-03637-9.

## Introduction

Cervical incompetence can lead to (extreme) preterm birth, defined as delivery at < 28 weeks of gestation [1]. A vaginal cerclage is effective to reduce the risk of recurrent extreme preterm birth in these circumstances [2]. In patients with a history of preterm birth, a vaginal cerclage significantly reduces the risk of preterm birth at 28, 34, and 37 weeks of gestation (average RR 0.77, 95% CI 0.66–0.89), resulting in a halving of the number of preterm births before 33 weeks [3]. In patients with a history of preterm birth and short cervical length, a cerclage reduces the incidence of immature delivery from 14.1% to 6.1% and perinatal mortality from 16% to 8.8% [4]. There is a significant reduction in the likelihood of preterm birth before 35 weeks in cases of cervical length < 15 mm (OR 0.23, 95% CI 0.08–0.66) [5]. The prevalence of history-indicated and ultrasound-indicated cervical cerclage is 1.19–1.91% [6, 7].

In patients with a failed vaginal cerclage, defined as a previous vaginal cerclage that resulted in delivery at < 28 weeks gestational age, an abdominal cerclage results in higher live birth rates [8]. An abdominal cerclage placed by laparoscopy or laparotomy can also be placed in patients with technical impossibility to place a vaginal cerclage for example after extensive cervical surgery, including (recurrent) loop electrosurgical excision procedure (LEEP), (laser) conization of the cervix and trachelectomy, although the additional value with regard to preterm birth rate in women with a LEEP or conization is not proven [8].

Suffering from extreme preterm birth and fetal loss puts an enormous strain on a patient and her family. Parents are at increased risk for anxiety, depression, post-traumatic stress disorder, and poorer overall well-being during and years after pregnancy [9–13]. This life event impacts a patient’s life both physically, emotionally, in social participation and on a personal level on multiple domains. The World Health organization (WHO) has defined these domains as body functions, daily life, participation, and personal factors in the International Classification of Functioning, Disability, and Health (ICF-DH). The ICF-DH is a framework that provides structured insight in functioning and limitations related to a health condition. It is a validated and commonly used tool in research to study patients’ perspective on functioning and disabilities [14]. Current research on vaginal and abdominal cerclages is mostly focused on surgical and obstetric outcomes [8, 15, 16]. Insights into patient perspectives by using the ICF-DH on their healthcare experience regarding preterm birth and management of pregnancy with a cerclage are lacking. The aim of this study is to assess the impact of a vaginal and abdominal cerclage on body functions, daily activities, participation, environmental, and personal factors according to the ICF-model and to evaluate their healthcare experience and needs.

## Material and methods

### Study methods

A qualitative focus group study including focus group discussion (FGD) was executed in participants with a vaginal and abdominal cerclage. For definitions of used concepts, see Appendix 1.

Prior to the FGD individual interviews were conducted by telephone to obtain a complete medical overview of baseline characteristics such as age and obstetric and gynecological history. Participants were asked to share their expectations regarding the FGD. An additional questionnaire (see Appendix 2) was executed to elucidate how participants experienced their pregnancy with cerclage including possible physical complaints, the received medical information and care. Results of these questionnaires were processed to a preset list (see Appendix 3) of complaints that could be experienced by participants after placement of a cerclage. A preset list of suggested improvements in healthcare in relation to patients with an extreme preterm birth and received cerclage was also processed. This preset list was used during the FGD for participants as an outline for potential topics to be discussed.

### Inclusion and data collection

This focus group study was performed at the department of Obstetrics and Gynaecology of the Amsterdam University Medical Center (Amsterdam UMC). Medical ethical clearance was obtained from the Medical Ethical Exam Committee of the Amsterdam University Medical Center, location Vrije Universiteit Amsterdam (020.460). Participants were recruited from our local database on LAC [16] or from medical records in the Amsterdam UMC. Participants were also recruited through the website of a Dutch patient organization for (extreme) preterm birth (www.extremevroeggeboorte.nl). After expressing their interest, participants were informed about the aim and procedure of the study by telephone and e-mail. All participants signed informed consent on paper and gave permission for audio recordings of the FGD. Anonymity and confidentiality were ensured. Eligible participants had full comprehension of Dutch language and access to internet. The latter was a requirement since physical meetings were not possible due to the COVID-19 pandemic. Eligible participants were ≥ 18 years old with a previous vaginal and/or abdominal cerclage and delivery at ≥ 34 weeks of gestational age with neonatal survival. We excluded participants who did not conceive after placement of an abdominal cerclage or participants who delivered at less than < 34 weeks of gestational age, because we considered it ethically insensitive to interview participants with good and poor obstetric outcomes in the same FGD. Besides, participants with positive obstetric outcomes may feel uncomfortable or inappropriate to share their experiences with respect to participants with adverse obstetric outcomes and vice versa. The FGDs were held in two separate groups; the vaginal and abdominal group since the indication, procedure and expected cerclage-related symptoms could be different.

We did not perform a sample size calculation but we predefined that we would continue with the FGD until no new information or insights emerged, indicating that a comprehensive understanding was gathered. In our study, we conducted data collection until no new themes or perspectives were emerging, and redundancy was observed in the information provided by the participants. We started with one FGD in each subgroup (vaginal and abdominal cerclage group) and aimed to include at least 8 participants in each group.

### Outcome measures

Outcomes were participants’ self-reported impact on their quality of life, encompassing physical, emotional, and social dimensions. Furthermore, experiences concerning received healthcare and their needs were assessed. Additional outcome measures included body functions and structures, activities, and participation and environmental and personal factors according to the Classification of Functioning, Disability, and Health (ICF-DH).

### Focus group discussions

**Fig. 1:**
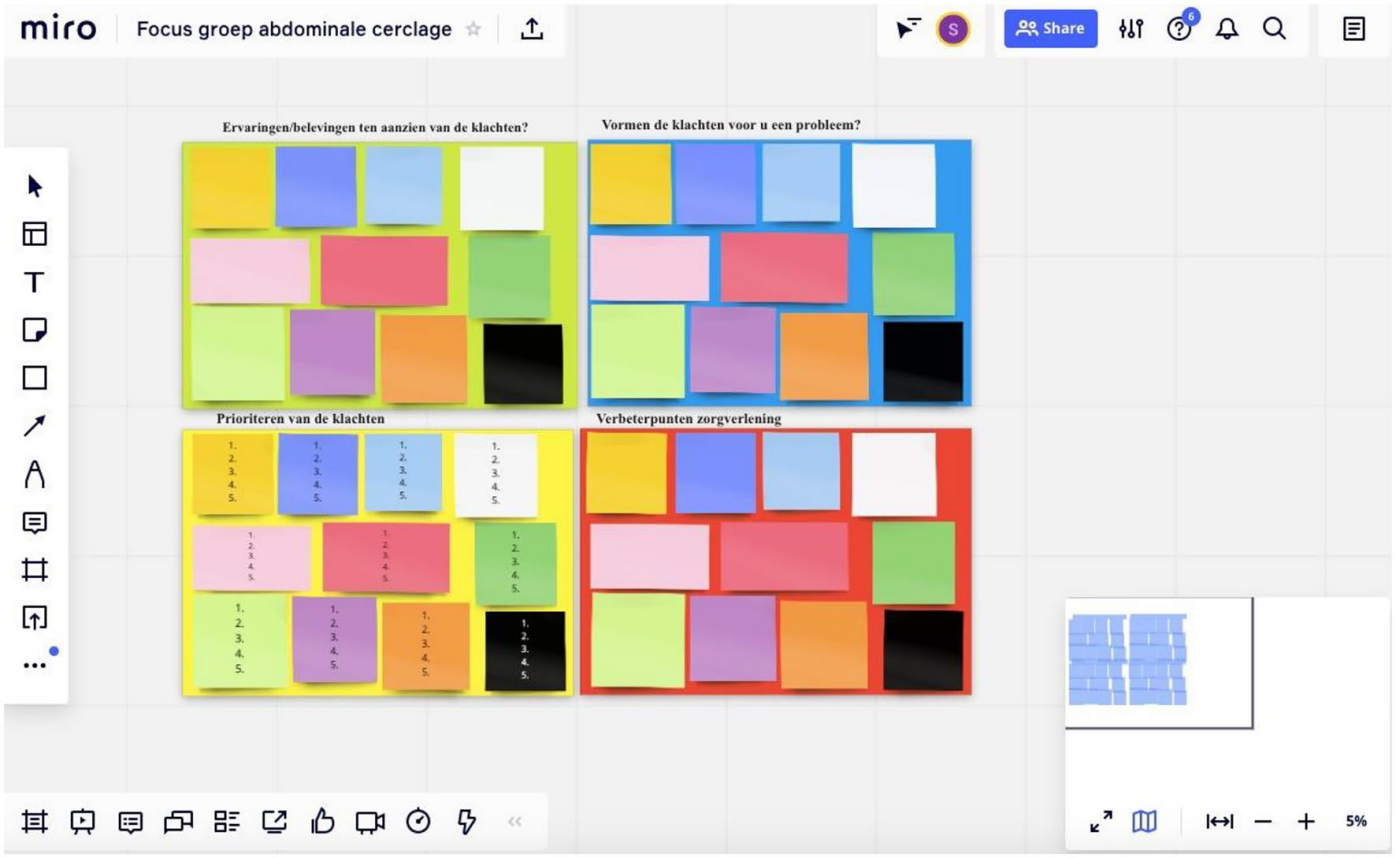
Miro online focus group whiteboard

The FGDs were conducted in January 2021. Microsoft Corporation Teams Office 365 version 1.3.00.30874 was used as an online and secure video conferencing platform. The online program Miro—a whiteboard platform enabling realtime visual collaboration using digital post-its—was used during FGD (see Fig. 1). All participants obtained access to the shared private document on Miro in advance and were designated to a post-it color to notate on during the FGD. Participants’ names were coded with their designated color to provide insight for the researchers into which participants gave which answers and to ensure privacy and shield personal information between participants. All FGDs were led by one experienced moderator (MBvZ) [17, 18].

A predefined format for the course of the FGDs was used in both groups. Details of the FGD are available in Appendix 4. In short, the format is as follows:

1. Introduction: it was explained that the purpose was to gain insight in outcomes related to functioning, disability, their healthcare experience, and their needs.
2. Extended information: participants were asked to write down complaints after cerclage placement and during subsequent pregnancy they had experienced in several domains based on a preset list.
3. Prioritizing: to explore the most limiting complaints participants were asked to individually prioritize their symptoms on the basis of level of experienced limitation.
4. Specific information: The impact of various complaints on daily, social, and sexual functioning after cerclage placement and during subsequent pregnancy was explored.
5. Healthcare experience: participants were asked to share their thoughts on their experienced healthcare and on their needs.
6. After the FGDs participants were asked to complete an evaluation form about their participation (see Appendix 6).

### Data analysis

**Fig. 2:**
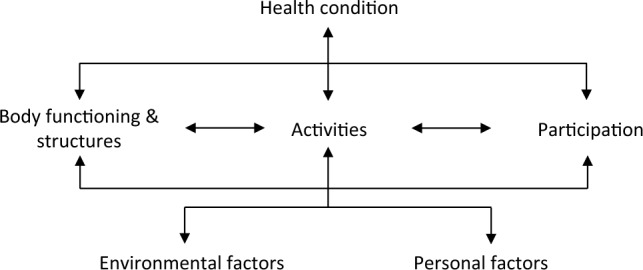
Interactions between the components of ICF

The FGD were transcribed verbatim with the use of the audio recordings and analyzed in Atlas.ti Scientific Software Development GmbH Version 8.4.24.0. The ICF-DH was used as an effective, systematic, and internationally recognized framework for statistical assessment and analysis [19]. It was used to provide a structured insight in functioning and limitations. Three multidimensional and interactive components classified in this ICF model are body functions and structures, activities and participation, and environmental and personal factors (Fig. 2).

**Fig. 3:**
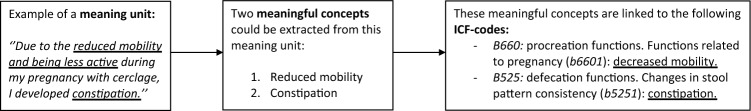
Visualization of coding procedure

To obtain an overview on functioning and complaints, coding of the transcripts was performed using the linking rule process [20]. Linking is a process that starts with carefully reading the transcripts. Subsequently, transcripts were divided into ‘meaning units,’ which are specific units of text, a few words, or sentences with a common theme. Each ‘meaning unit’ was linked to a dominating theme, a so-called ‘meaningful concept.’ One ‘meaning unit’ can contain more than one ‘meaningful concept.’ ‘Meaningful concepts’ were divided over three components of the ICF model: (i) body functions and structures, (ii) activities and participation, and (iii) environmental and personal factors, and were linked to the corresponding ICF codes. An example is shown in Fig. 3.

The frequencies of the identified concepts were calculated and reported. The ICF codes were complemented with information, experiences, and views of the participants. The relevant codes were entered into Table 1 (with an extended version in Table 5, see Appendix 5), to summarize the data and provide a structured overview of the linked ICF codes per ICF component.

**Table 1:**
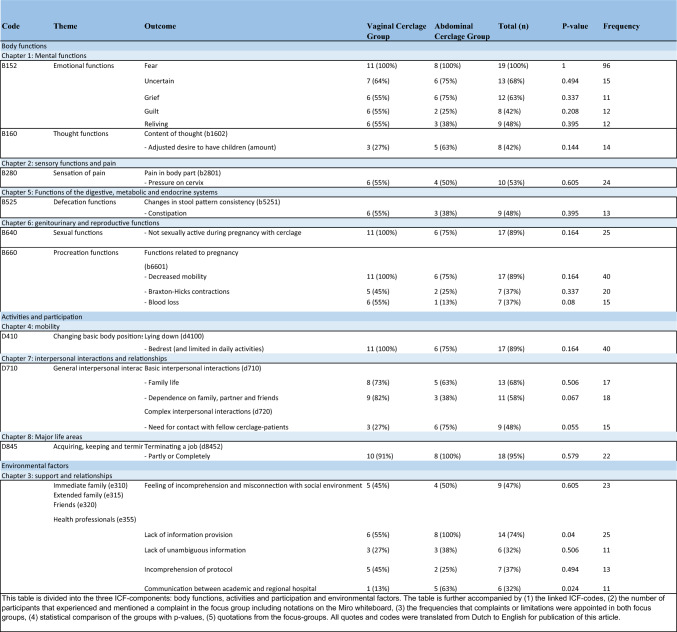

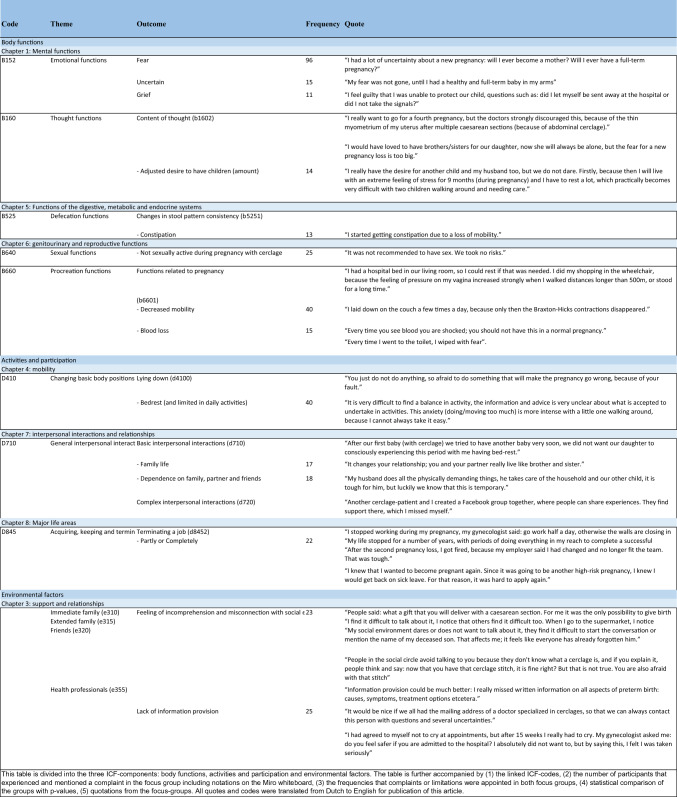
Patient perspectives

### Statistical analyses

Statistical analyses were conducted using IBM SPSS Statistics, version 26.0 (IBM Corp., IBM SPSS Statistics for Windows, Version 26.0. Armonk, NY, USA). Descriptive statistics were used to summarize baseline characteristics. A Shapiro–Wilk test was performed to determine normal distribution of data. To compare paired data with normal distribution a paired t-test was used. In case of non-normal distribution, a Wilcoxon signed-rank test was performed. For unpaired data with normal distribution, an unpaired t-test was used. If the assumption of normality was violated, a Mann–Whitney U test was performed. Fisher’s exact test was used to compare categoric variables. All tests were 2-sided; a probability value of < 0.05 was considered significant.

## Results

**Fig. 4:**
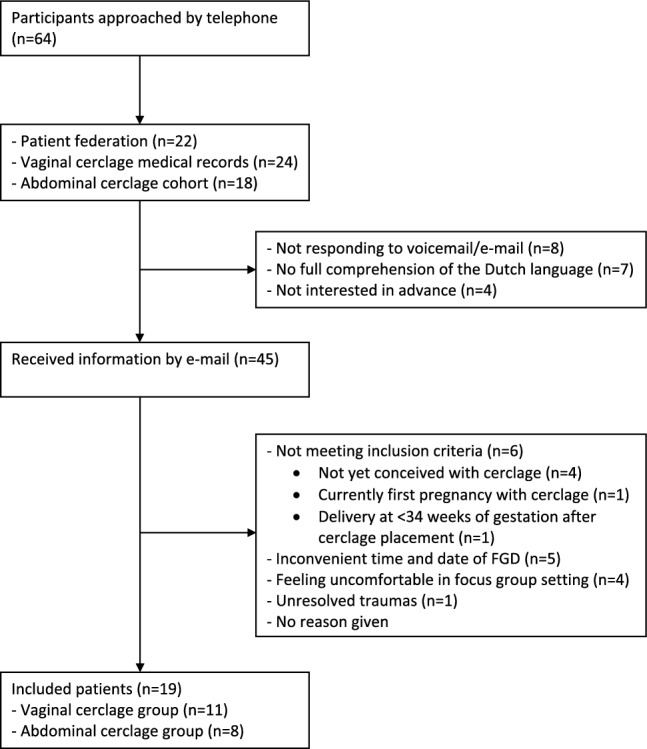
Flowchart patient inclusion

A total of 64 eligible participants were identified; 18 participants from the Amsterdam UMC LAC database, 24 participants with a vaginal cerclage from medical records, and 22 respondents from the patient organization. Of these 64 participants, 19 participants did not respond to telephone and e-mail (*n* = 8) or did not have full comprehension of the Dutch language (*n* = 7) or were not interested (*n* = 4). The remaining 45 participants received the participant’s information letter. Twenty-six participants could not participate, the majority of them without reason (*n* = 10) and others because the time and date of the scheduled FGD meetings were inconvenient (*n* = 5). Nineteen participants were included; 11 participants in the vaginal cerclage group (VCG) and 8 participants in the abdominal cerclage group (ACG) (see Fig. 4). In both groups, data saturation was achieved so it was not needed to schedule additional FGD meetings.

**Fig. 5:**
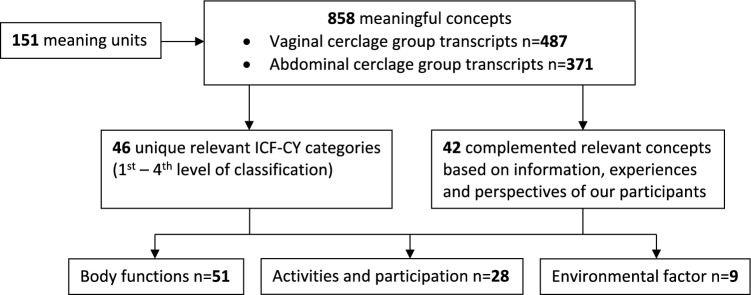
Flowchart of the code-linking procedure

The code-linking procedure of the FGD with identification of meaning units and meaningful concepts is visualized in Fig. 5.

### Demographic results

Mean age was 36,2 ± 4,4 in the VCG and 38,5 ± 4,9 in the ACG. Participants in the ACG had more second- and/or third-trimester fetal losses in their medical history compared to the VCG (median ACG 2,0 ± 1,8, VCG 1,0 ± 1,0, *p* = 0,05). Significantly more participants in the ACG (62,5%) had more than two second- and/or third-trimester fetal losses in history compared to the VCG (9,1%). Other demographic results such as gravidity and parity did not differ significantly between both groups. All participants’ characteristics are summarized in Table 2.

**Table 2:**
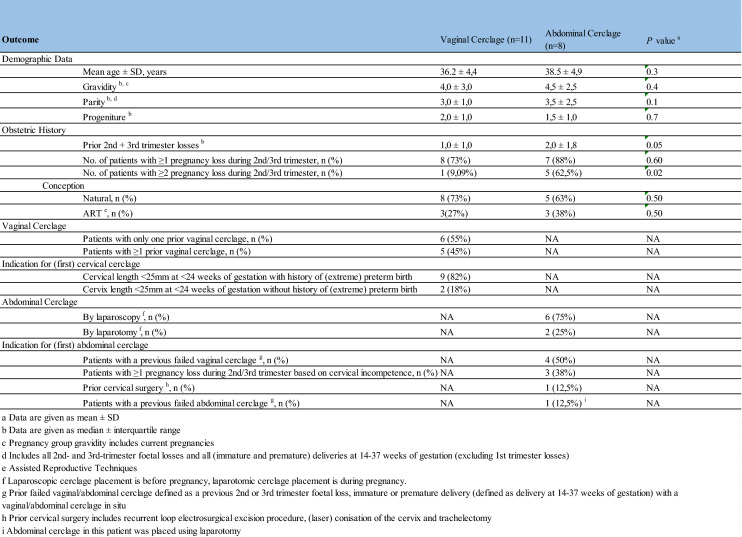
Baseline characteristics

### Patient questionnaire (prior to focus group discussions)

In both the VCG and ACG 63% were satisfied with the cerclage-related information prior to placement received by their healthcare provider. Two participants (18%) in the VCG who received a tertiary vaginal cerclage in retrospect felt insufficiently informed about the surgical procedure. Two participants (25%) in the ACG reported that in retrospect they received insufficient information about possible complications in relation to cerclage placement or during subsequent pregnancy. Five participants (63%) in the ACG indicated that they would rather have been informed earlier about the possibility to receive an abdominal cerclage since they experienced multiple second-trimester fetal losses and/or preterm births despite a vaginal cerclage. The majority of all participants used internet and patients forums (VCG = 73%, ACG = 88%) to gain additional information about the indication, procedure of placement, possible risk and complications, and success rates during subsequent pregnancy with cerclage. For details see Table 3.

**Table 3:**
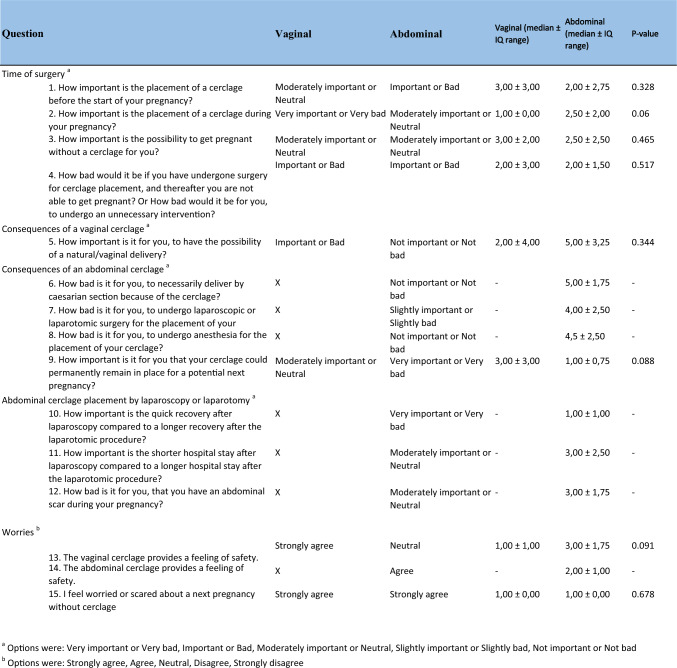
Patient questionnaire

### Patient perspectives

All participants expressed fear for complications during a subsequent pregnancy such as pregnancy loss and preterm birth as most limiting problem in performing activities in daily life.

Participants mentioned: “*I had a lot of uncertainty about a new pregnancy: will I ever become a mother? Will I ever have a full-term pregnancy?.”*

Fear to conceive because of fear for a recurrent adverse pregnancy outcome as a consequence of poor obstetric history was experienced by 27% in the VCG and 13% in the ACG before placement of a cerclage.

One participant mentioned: *“I really have the desire for another child and my husband too, but we do not dare. Firstly, because then I will live with an extreme feeling of stress for 9 months and I have to rest a lot, which practically becomes very difficult with two children walking around and needing care.”*

The anxiety for complications during their subsequent pregnancy also negatively impacted their physical activities. Despite the cerclage placement 55% in the VCG and 25% in the ACG were afraid that their physical activity would contribute to an adverse outcome. The majority did experience anxiety reduction after placement of the cerclage, this was 64% in the VCG and 75% in ACG.

#### Body functions during pregnancy

Participants in the VCG (91%) reported on physical complaints, such as Braxton-Hicks contractions and vaginal blood loss. This was reported in 50% in the ACG participants. The main reported complaint in both groups was cervical pressure (VCG = 55%, ACG = 50%).

One participant in the VCG mentioned: “*I had a hospital bed placed in our living room, so I could rest whenever needed. I did my shopping in a wheelchair, because the feeling of pressure on my vagina increased strongly when I walked distances longer than 500 m, or when I stood on my legs for a long time.”*

Reduced mobility was reported by all participants in the VCG and by 75% in the ACG. None of the participants in the VGC dared to have sexual intercourse compared to 63% in the ACG. Some participants were recommended by their healthcare provider to reduce mobility and avoid sexual intercourse during pregnancy.

One respondent in the VCG mentioned: *“It was recommended to not have sex. We took no risks.”*

#### Activities and participation

The fear for complications, the physical complaints, and recommended bed rest during pregnancies prevented participants from engaging in physical and social activities. The feeling of social isolation was mentioned in 18% in the VCG and 25% in the ACG.

One participant mentioned: *“I did not do anything, I was so afraid to do something that might contribute to an adverse outcome of the pregnancy, because of my fault.”*

All participants were advised by their healthcare providers to decrease pressure on the cervix by avoiding lifting, heavy domestic work, walking long distances and stand for a long period of time. This led to a dependency on family and friends during pregnancy, 82% in the VCG and 38% in the ACG group.

One respondent mentioned: *“My husband does all the physically demanding things, he takes care of the household and our other child, it is tough for him, but luckily we know that this is temporary.”*

Nearly all participants (VCG = 91%, ACG = 100%) partially or completely stopped working from 14 weeks of pregnancy onwards (frequently advised by their healthcare providers), which also affected their social participation. This inability to work led to a sense of guilt and embarrassment towards employers and colleagues.

One participant mentioned: *“My life stopped for a number of years, with periods of doing everything in my reach to complete a successful pregnancy, to physical and mental recovery, to be able to work again for a while, and then this circle repeated. This was disastrous for my career.”*

#### Environmental factors

Participants lacked the feeling of connecting to their inner social circles and experienced difficulties in communicating about their condition and emotions with others (VCG = 45%, ACG = 50%). Two outcomes regarding ‘social support’ were identified: (1) acceptance: cerclage-related symptoms and the loss of a pregnancy/child are considered to be a taboo as social contacts avoided talking about it, (2) incomprehension: others were unfamiliar with cerclages and they could not relate to the situation, which was considered as burdened.

One participant mentioned: *“People in the social circle avoid talking to you because they don’t know what a cerclage is, and if you explain it, people think and say: now that you have that cerclage stitch, it is fine right? But that is not true. You are also afraid after that stitch.”*

#### Prioritizing

The following top four complaints were prioritized in both groups: fear of a subsequent complicated pregnancy and stress during pregnancy, decreased mobility, uncertainty/helplessness/loss of control, feeling of pressure in the vagina and lower abdomen. In addition, in the VCG the feeling of dependency on other people/family and in the ACG insufficient knowledge and lack of information provided by healthcare professionals in the secondary care was mentioned as top five complaint. See Table 4 for a detailed overview of all complaints mentioned during the prioritizing process.

**Table 4:** Prioritizing, top five complaints

Complaint	Frequency
** Vaginal Cerclage Group (n = 11)**	
Fear	11
Dependency	8
Decreased mobility	8
Uncertainty/loss of control	5
Feeling of pressure in the vagina and lower abdomen	5
Feeling of guilt about not being able to care for family during pregnancy	3
Loneliness	2
Sadness	2
Reliving of previous pregnancies/deliveries	2
Difficulties in connecting with others	2
Difficulties with functioning at work	2
Small focus	1
Braxton-Hicks contractions	1
Constipation	1
Loss of mucus	1
** Abdominal Cerclage Group (n = 8)**	
Fear and stress	8
Immobility	6
Insufficient knowledge and lack of information provided by healthcare professionals	5
Pressure/pain on cerclage	4
Feeling of helplessness/loss of control	4
Constipation	3
Feeling of incomprehension	2
Social isolation	2
Fear for caesarean section	1
Braxton-Hicks contractions	1
Tiredness	1
Dyspareunia	1
Discomfort after laparotomic cerclage	1
Waiting list for cerclage placement	1
Not being able to enjoy pregnancy	1

#### Healthcare experience

The lack of consistent information regarding management and expectations during pregnancy mainly by gynecologists from tertiary hospitals was mentioned in 27% and 38% in the VCG and ACG, respectively. In secondary care hospitals the lack of clear, coherent, and general information mainly by obstetricians about the cerclage itself, including expectations during pregnancy and instructions given on their daily, work, and sexual activity was expressed by 55% and 100% in the VCG and ACG, respectively.

Participants experienced a lack of knowledge concerning the abdominal cerclage in secondary care hospitals. Communication between tertiary and secondary care hospitals was mentioned as point of improvement by half of the participants in the ACG. Participants received inconsistent information on the need for cervical length measurements, bedrest, and sexual prohibition between tertiary and secondary care hospitals. Participants expressed a strong need for a patient information leaflet providing unambiguous information.

One participant mentioned: *“Information provision could be much better: I really missed written information on all aspects of preterm birth: causes, symptoms, treatment options etcetera.”*

The majority of the participants (63%) in the ACG expressed that they had received unclear counseling pre-operatively about the success rates and expectations during pregnancy with abdominal cerclage from mainly obstetricians in tertiary care hospitals.

One participant mentioned: *“It would be nice if we all had the mailing address of a doctor specialized in cerclages, so that we can always contact this person with questions and several uncertainties.”*

The importance of contact with fellow cerclage patients was mentioned by 27% in the VCG and 75% in the ACG. All participants were members of online patients forums or Facebook pages to get in contact with other patients with a cerclage. Additional psychologic support was desired in half of the participants in both groups.

#### Evaluation of the focus groups

Participants experienced support in the FGD (VCG = 64%, ACG = 63%).

*‘’It was nice to participate in the focus groups, because I recognized myself in the story of others and it made me feel less alone. It also gave me the opportunity to share my story with others. However, it was also difficult to hear the sad stories of others.’’*

Participants reported that they felt comfortable sharing their own experiences (VCG = 73%, ACG = 100%) and felt less alone after participating in the FGD (VCG = 64%, ACG = 75%). Participants in both groups recognized experiences of other patients, median score of recognition on scale 0–10 was 8 and 7,5 in the VCG and ACG, respectively.

## Discussion

To our knowledge this is the first study that by means of focus group discussion evaluated the impact of a cerclage on daily functioning and patient perspectives on their healthcare experience and their needs regarding care in patients with a high risk of (extreme) preterm birth and an indication for cerclage. Fear for a subsequent complicated pregnancy before and after conception was mentioned as the most limiting factor, it had a very strong negative impact on daily functioning, participation, quality of life, and social and sexual functioning. Comprehensive and unambiguous information about the success rate of an abdominal cerclage and potential complications was missing (VCG = 27%, ACG = 63%). According to participants information about obstetric management following cerclage from caregivers in different hospitals was unclear and varied widely (VCG = 55%, ACG = 100%). In particular information on the value of cervical length measurements by ultrasound, value of tocolysis, the need for bedrest, daily work, and sexual activity was missing in both groups. In relation to this, it is worth noting that cervical length measurement after cerclage placement has no additional value [21, 22]. In addition, there is no substantial evidence that the use of tocolysis during cerclage placement for elective or ultrasound-indicated vaginal cerclage results in an improved outcome. However, additional research with regard to physical activity and sexual activity is needed for a more evidence-based advice. In our study, the impact of bedrest or restriction of activities on psychological and social well-being is substantial. Yet, studies have demonstrated that bedrest does not significantly prolong pregnancy including for women with short cervices and ruptured membranes [23, 24]. It has been shown that physically demanding work including occupational shift and night work increase the risk of preterm birth [25–27]. Therefore, in most guidelines regarding women with a high risk of preterm birth, it is recommended to reduce physical activity. However, strict bedrest does not seem to be of additional value and this should be emphasized to women.

This study shows that there is a clear demand for unambiguous information for patients and management of expectations in the form of patient information leaflet for both groups. Furthermore, participants reported on a strong wish for psychological aftercare and support. Referral to psychologists with expertise in the field of obstetrics is recommended, which could contribute in reducing anxiety and stress [28, 29]. Participants in our study clearly expressed the need for peer support with fellow cerclage patients. Patients want to share their experiences and improves empowering and knowledge. A possibility is to facilitate this through the website of the Dutch Society of Obstetrics and Gynaecology and efforts are currently taken to provide information on cerclages and advice regarding lifestyle.

Physical complaints during pregnancy after cerclage placement such as feeling of pressure in the vagina, dyspareunia, and blood loss were experienced more frequently after vaginal than after abdominal cerclage placement. This can be explained by the fact that an abdominal cerclage is placed higher and closer to the uterine corpus than a vaginal cerclage resulting in a different pressure distribution. An abdominal cerclage is therefore expected to provide more mechanical support to the cervix and to reduce the risk on funneling and therefore potentially results in reduced feeling of pressure. This is in line with the fact that an abdominal cerclage is more effective than a vaginal cerclage in patients with poor obstetric history [8]. However, it is important to emphasize that our study was of qualitative nature and results were not quantitatively analyzed in this study. Furthermore, we acknowledge the limitation stemming from the conduct of one FGD in the VCG and one FGD in the ACG. While we determined that no new information or insights emerged in these FGDs, we acknowledge that conducting more FGDs in each category could have enhanced the robustness of our data by capturing a broader range of perspectives. It is noteworthy that despite reaching out to 64 potential participants, we successfully motivated 19 individuals to participate in our study. This emphasizes the challenges and barriers to participation. While we ideally acknowledge that multiple focus groups would have strengthened the study, practical constraints in daily practice made this unfeasible.

Further research is needed to assess the best obstetric management for patients with an abdominal cerclage by performing a prospective cohort study on the value of monitoring, application of progesterone, and prevention of lifting and extensive physical activities in this group. Women should be informed that they do not need to take bedrest or restriction of normal daily activities. The value of routine measurement of cervical length measurement after cerclage placement in asymptomatic women is debatable. As it may predict patients at a higher risk for preterm birth, no additional treatment options are available. In patients with a cerclage and symptoms of preterm labor, measurement of residual cervical length may be of value in differentiating patients in actual preterm labor and those with false symptoms. However, a normal cervical length in patients with an abdominal cerclage may be misinterpreted as a low risk for a delivery in short term. Currently, we are studying the value of assessing the position of an abdominal cerclage prior to a (subsequent) pregnancy.

One of the strengths of our study is that we applied a non-directive method to ask the input of participants to decrease the threshold to provide input and share their experiences. Also the FGD were coordinated by an independent professional in order to prevent any influencing of the answers or preventing socially desirable answers of the participants. Another strength was the use of the ICF model to provide a holistic view of participants, assess complexities of functioning, analyze data in a unified language, and have an insight into functioning. The ICF model fitted our data well, as many ICF themes on functioning were mentioned in our focus group discussions. However, one of the limitations of this study is that a lot of categories of the ICF model were not supported by gynecological symptoms. These were complemented in this study with the experiences of participants. Future research is necessary to create an ICF core set for the field of obstetrics and gynecology.

Our pre-designed interview scheme with ICF-approach helped the coordinator and participants to be as complete as possible. It gave the opportunity for participants to mention experiences and problems in all possible domains including functioning, which could be too sensitive to mention spontaneously in an open approach. The discussion of sensitive topics was also stimulated by using the online whiteboard platform, where participants were able to write down their complaints without saying it out loud.

A deliberate choice in study design was the inclusion of participants with delivery > 34 weeks of gestation after cerclage placement only. This is a limitation of our study since it has created selection bias, which probably results in more positive outcomes with regard to the psychological domain. However, interviewing participants with good and poor obstetric outcomes in one focus group might result in information bias as participants in both groups might not feel comfortable sharing their story. In order to provide information on the experiences of patients with failed cerclage, further research and additional focus group discussions are required. This might be difficult for failures (i.e., second-trimester fetal loss or extreme preterm birth) after an abdominal cerclage since the total number of failures so far is extremely low [16, 30].

Two participants also suffered from other gynaecologic conditions (endometriosis, fibroids, and uterine niche), which may have interfered with specific complaints. We asked these two participants to report only on the gynecological complaints that started or exacerbated after cerclage placement, but any overlap of complaints cannot be excluded.

## Conclusion

In conclusion, in this focus group study fear of a subsequent adverse pregnancy outcome was the most frequently reported and most limiting complaint in daily life after both vaginal and abdominal cerclage placement. The placement of a cerclage contributes to anxiety reduction in the majority of participants. Physical complaints were experienced more frequently in the VCG compared to the ACG. Increasing awareness and understanding of emotional needs and psychological support in patients with a cerclage is a clear point of improvement. We observed strong adverse effects on physical, emotional, and social well-being as a result of strict bedrest advice. Unfortunately, no high level evidence is available on this matter. Patients might even benefit from appropriate levels of physical activity throughout their pregnancy to promote their overall well-being. Physically demanding work including shift and night work should be avoided, as this might increase the risk of preterm birth. More evidence is needed to determine the optimal level of physical activity. Unambiguous information about obstetric management following cerclage, both in tertiary and secondary hospitals, towards patients is desired, since participants report discrepancies between hospitals and caregivers. It is recommended to provide a patient information leaflet.

### Supplementary Information

Below is the link to the electronic supplementary material.

Supplementary file1 (DOCX 48 KB)

## Data Availability

The data supporting the findings of this study are available within the article and its supplementary materials.

## Abbreviations

FGD: Focus Group Discussion
VCG: Vaginal Cerclage Group
ACG: Abdominal Cerclage Group
WHO: World Health Organization
ICF-DH: International Classification of Functioning, Disability and Health
ICF: International Classification of Functioning
LAC: Laparoscopic Abdominal Cerclage
LEEP: Loop Electrosurgical Excision Procedure
Amsterdam UMC: Amsterdam University Medical Center
