# Supplementary material for: Patient perspectives and preferences on cerclage and preterm birth: a focus group study
Source: Qual Life Res. 2024 Jun 18;33(8):2165–79. doi: 10.1007/s11136-024-03637-9 (PMC11286660; doi:10.1007/s11136-024-03637-9)
Supplement: Supplementary file 1 — Supplementary file1 (DOCX 48 KB) [file 11136_2024_3637_MOESM1_ESM.docx]

- 1. Appendices

**Appendix 1: Definitions**

A failed vaginal cerclage is defined as a previous vaginal cerclage that resulted in delivery at <28 weeks of gestation. (8) Extreme preterm birth is defined as delivery at less than 28 weeks gestational age. (1) Prior cervical surgery includes (recurrent) loop electrosurgical excision procedure (LEEP), (laser) conisation of the cervix, and trachelectomy. (8) Primary cerclage was defined as placement of a cerclage before cervical changes (prophylactic) (31), secondary cerclage as placement of a cerclage after cervical shortening (therapeutic) (32), and tertiary cerclage as placement of a cerclage after cervical dilation and/or exposure of membranes to the vagina (therapeutic rescue cerclage). (33)

**Appendix 2: Patient Questionnaire**

**Patient Questionnaire**

| 1. How did you experience the counselling/information provision about the surgical procedure (placement of a stitch around the cervix)?   _________________________________________   1. In addition to the vaginal cerclage, have you also received information about the abdominal cerclage?    - Yes   If so, what was the main reason for you to choose your type of cerclage (vaginal or abdominal)?   - The possibility of placement of the cerclage before pregnancy; - The possibility of placement of the cerclage during pregnancy; - The need for a caesarean section with an abdominal cerclage; - The reduced risk of recurrent miscarriage/fetal death; - The choice of type cerclage was decided for me, I had no choice.   - No  1. Have you been sufficiently informed about the surgical procedure (placement of a suture around the cervix)? If you have had both a vaginal and an abdominal cerclage, please note your answer for both types of cerclage.    - Yes, I am sufficiently informed;    - No, I do not feel sufficiently informed. Would you like to indicate which information you have missed;   _________________________________________   - - Other, namely   _________________________________________   1. How were you informed about the possibility of an abdominal cerclage (multiple answers are possible)?    - by my gynecologist    - by other healthcare providers such as;    - my midwife    - Other, namely | - - my doctor/general practitioner   - other, namely***   - by family/friends   - by websites/forums   - not applicable/not informed   - other, namely ___________  1. Would you rather have been informed earlier about the possibility of an abdominal cerclage? If so, at what time would you like to be informed?  - Yes - No - Not applicable   _________________________________________   1. In addition to information from your gynecologist, what sources of information have you used to help you make a decision about whether or not to have a vaginal or abdominal cerclage (multiple answers are possible)  - None - Books/magazines - Family/friends - Websites/forums - Other health care providers, such as; - My midwife - My doctor/general practitioner - Other, namely ________ |
| --- | --- |

**Appendix 3: Preset list of outcomes**

| 1. **Physical complaints**    - Gynecological  \| **During pregnancy:**  Blood loss   - - - - After cerclage placement   (cloudy or excessive) loss of mucus  Pain   - - - Vagina     - Lower abdomen     - Pressure cervix   Fever/infections  Fatigue  Decreased mobility  Braxton Hicks contraction  PPROM \| **Not during pregnancy:**  Blood loss  (cloudy or excessive) loss of mucus  Pain   - - - Vagina     - Lower abdomen     - Pressure cervix   Fever/infections  Fatigue  Decreased mobility \| \| --- \| --- \|  - - Urogynecology     - Pain with full bladder     - Pain after micturition     - Frequent micturition   - Other physical complaints     - Changes in stool pattern     - Changes in menstruation cycle  1. **Sexual complaints**    - Sexual problems      - Blood loss after sex      - Pain      - Less sex drive      - Shame    - Problems with partner      - Less sexually active      - Feeling of shame/guilt against partner    - Fertility      - Fear to become pregnant again 2. **General health**    - Less energy    - Feeling less fit    - Social problems    - Changes in focus/attention    - Changes in thoughts    - Changes in self esteem    - Changes in relationship with partner    - Changes in relationship with family    - Changes in relationship with friends    - Feeling of incomprehension in social circles    - Feeling of incomprehension by health care providers    - Feeling of incomprehension by institutes    - Emotional or physiological complaints      - 1. Self esteem        2. Fear        3. Grief        4. Loss of control        5. Helplessness        6. Reliving        7. Guilt        8. Loneliness        9. Sadness        10. Shame        11. Uncertain        12. Unfulfilled child wish        13. other 3. **Social-cultural participation problems**     - Limitation in daily activities    - Limitation in social activities, like friends and family    - Less functioning at work and absenteeism |
| --- | --- | --- |

**Appendix 4: Extended information on the focus group discussion**

| **Extended information on the execution of the focus groups.**   1. *Introduction*: the focus groups started with an introduction. The moderator and researchers were introduced by name and function. The moderator explained the procedure and purpose of the focus group study. Patients provided their obstetrical history and motivation to participate in this study. 2. *Extended information*: To gain insight in experienced problems of patients with a cerclage or prior preterm birth, the moderator asked each patient to write down their experienced complaints in several domains. These domains were based on a preset list, comprising various outcomes. This list was made in advance based on clinical practice, the individual interviews and the ICF-categories. This list consisted of 8 categories, which were as follows: Gynecological symptoms, urogynecology symptoms, other physical symptoms, sexual health, general health, emotional and psychological symptoms and participation problems. The patients noted the outcomes on this preset list which they recognized or experienced in the period after their cerclage placement. This provided a broad range of outcomes, which were listed at the Miro site^23^. Miro is an online collaborative whiteboard platform where all the patients could sign in and interactively add and adjust texts at the same time. All patients gained access to this shared private document in advance and were designated to a post-it color to notate in during the focus groups. This shared online whiteboard with post-its, provided a structured and clear overview of experienced problems by the patients. All noted outcomes were collectively and elaborately discussed in detail. The group exchanged views about the recognition and limitations of the appointed symptoms. The most limited complaints on the preset list were collectively checked for relevance and recognition with leading of the moderator. The use of this approach, to check the symptoms on the preset-list one-by-one, provided an opportunity to gain insight in the frequency of certain problems in patients with a cerclage. The number of times that a symptom was mentioned or notated in the focus groups was noted and elaborated for further analysis. 3. *Prioritizing*: To explore most limiting complaints, the patients were asked to prioritize the experienced symptoms on the basis of severity. The prioritization list was appointed 1 to 5, and ranked the most affecting symptom at number 1, the second most affecting at number 2, etc. A total relevance score was made for all outcomes and domains, concerning the 1 to 5 priority list. The outcomes were summarized in a general top five list, ranked to importance of patient’s averages. 4. *Specific information*: To gain more specific information on the prioritized symptoms, the impact of various complaints was identified. This gave the opportunity to explore the effect of the symptoms on daily life of the patients. Several aspects, such as physical limitations, emotional impact, relational effect, fertility problems, difficulties to talk about the subject, feeling misunderstood and fear for a subsequent pregnancy, were discussed. Extra attention was drawn to the impact of a pregnancy loss, and the potential anxiety reduction after the cerclage placement. 5. *Care provision process*: patients were asked to share their experiences on the cerclage care process during the focus groups. The current health care provision regarding cerclages was examined and assessed in order to improve patient-centered care. Patients talked about their own care procedure and the corresponding points of improvement they observed. The moderator stimulated engagement by discussing especially topics as: Referral, diagnostics, guidance and check-ups, treatment, relationship with the health care professional, therapies and follow-up care. |
| --- |

**Appendix 5: Table 5, supplement to table 1, extended table with limitations appointed in the focus group discussions**

| Code | Theme | Outcome | **Vaginal group (n)** | **Abdominal group (n)** | **Total (n)** | **P-value** | **Frequency** | **Quote** |
| --- | --- | --- | --- | --- | --- | --- | --- | --- |
| **BODY FUNCTIONS** | | | | | | | | |
| **Chapter 1: Mental functions** | | | | | | | | |
| B140 | Attention functions | Trouble in keeping and dividing attention (b1400 and b1402) | 3 (27%) | 0 (0%) | 3 (16%) | 0,170 |  | *“In my social circles people said: go read a nice book and watch a movie, but if you have that extreme fear, you don’t enjoy reading a book or watching a movie.”*  *“I really needed my job to clear my mind sometimes.”*  *“I had a lot of concentration problems at that time.”* |
| B130 | Energy and drive functions | Energy level (B1300) | 2 (18%) | 4 (25%) | 6 (32%) | 0,319 |  | *“I had less energy, because I was not allowed to move, for me this was no limitation: I would have done everything for a “full-term” pregnancy.”* |
| B152 | Emotional functions | Fear  Uncertain  Grief  Guilt  Reliving  Loss of control  Helpless/powerless  Loneliness or depression  Sadness | 11 (100%)  7 (64%)  6 (55%)  6 (55%)  6 (55%)  7 (64%)  3 (27%)  3 (27%)  4 (36%) | 8 (100%)  6 (75%)  6 (75%)  2 (25%)  3 (38%)  1 (13%)  4 (50%)  1 (13%)  1 (13%) | 19 (100%)  13 (68%)  12 (63%)  8 (42%)  9 (48%)  8 (42%)  7 (37%)  4 (21%)  5 (26%) | 1  0,494  0,337  0,208  0,395  0,037  0,297  0,426  0,267 | 96  15  11  12  12 | *“My fear was not gone, until I had a healthy and full-term baby in my arms”*  *“I had a lot of uncertainty about a new pregnancy: will I ever become a mother? Will I ever have a full-term pregnancy?”*  *“I feel guilty that I was unable to protect our child, questions such as: did I let myself be sent away at the hospital or did I not take the signals?”*  *“After our second loss, I fell into a severe depression, I was very afraid this would happen again after a new pregnancy loss.”* |
| B160 | Thought functions | Content of thought (b1602)  - Adjusted desire to have children (amount)  - Counting pregnancy terms and living from day-to-day  - Not able to enjoy being pregnant | 3 (27%)  2 (18%)  3 (27%) | 5 (63%)  2 (25%)  2 (25%) | 8 (42%)  4 (21%)  5 (26%) | 0,144  0,574  0,664 | 14 | *“I really want to go for a fourth pregnancy, but the doctors strongly discouraged this, because of the thin myometrium of my uterus after multiple caesarean sections.”*  *“I would have loved to have brothers/sisters for our daughter, now she will always be alone, but the fear for a new pregnancy loss is too big.”*  *“I lived from appointment to appointment and from milestone to milestone.”*  *“You are scared to look forward and really live from day-to-day. From around 30 weeks we dared to be more positive and started furnishing the baby room and started buying some baby clothes.”*  *“It was very difficult to bond with my unborn child and enjoy being pregnant.”  “After the cerclage was removed at 37 weeks, I could be pregnant without any worries, it felt amazing!”* |
| B180 | Experience of self and time | Confidence yourself and in your body (b1800 and b1801) | 4 (36%) | 4 (50%) | 8 (42%) | 0,449 |  | *“Every miscarriage feels like failure of me as a woman, of my body. Any women should be able to do this (having a full-term pregnancy), even though I unfortunately knew better.”*  *“All the faith in my body was gone, and it is very, very difficult to rely on a string (cerclage) to keep everything together down there.”*  *“I was angry and disappointed in my body for a long time.”* |
| **Chapter 2: sensory functions and pain** | | | | | | | | |
| B280 | Sensation of pain | Pain in body part (b2801)  - Pressure on cervix  - Rupture of cervix  - Vagina/Lower abdomen (b28012) | 6 (55%)  1 (9%)  3 (27%) | 4 (50%)  1 (13%)  2 (25%) | 10 (53%) | 0,605  0,177  0,664 | 24 |  |
| **Chapter 5: Functions of the digestive, metabolic and endocrine systems** | | | | | | | | |
| B525 | Defecation functions | Changes in stool pattern consistency (b5251)  - Constipation | 6 (55%) | 3 (38%) | 9 (48%) | 0,395 | 13 | *“I started getting constipation due to a loss of mobility.”* |
| **Chapter 6: genitourinary and reproductive functions** | | | | | | | | |
| B620 | Urinary functions | Changes in micturition pattern  - Polyuria (b6201)  - Painful micturition  - Infection  - Incontinence (b6202) | 2 (18%)  1 (9%)  0 (0%)  2 (18%) | 3  0  1  2 | 5 (26%)  1 (5%)  1 (5%)  4 (21%) | 0,336  0,579  0,421  0,574 |  | *“Since my last pregnancy I cannot control it (urine incontinence), especially when getting up in the morning, I smell something that I should not smell.”* |
| B640 | Sexual functions | - Not sexually active during pregnancy  - Dyspareunia  - Decreased sex drive (b6400)  - Bleeding during/after sex  - Feeling of guilt (decreased sexual activity) | 11 (100%)  0 (0%)  0 (0%)  1 (9%)  2 (18%) | 6 (75%)  2 (18%)  2 (18%)  1 (9%)  0 (0%) | 17 (89%)  2 (11%)  2 (11%)  2 (11%)  2 (11%) | 0,164  0,164  0,164  0,678  0,322 | 25 | *“It was not recommended to have sex. We took no risks.”*  *“After childbirth I had pain during intercourse, which caused less sex drive. That is annoying for both of us.”*  *“I sometimes feel the cerclage during intercourse.”*  *“During ovulation I am extra alert with having sex. We now use contraception (condom) and I am very strict about this, especially because I am afraid of a pregnancy.”* |
| B660 | Procreation functions | Functions related to pregnancy  (b6601)  - Decreased mobility  - Braxton-Hicks contractions  - Blood loss  - Fatigue  - Pelvis floor issues  - Loss of mucus  - Infection/fever | 11 (100%)  5 (45%)  6 (55%)  2 (18%)  7 (64%)  2 (18%)  0 (0%) | 6 (75%)  2 (25%)  1 (13%)  4 (50%)  2 (25%)  0 (0%)  0 (0%) | 17 (89%)  7 (37%)  7 (37%)  6 (32%)  9 (47%)  2 (11%)  0 (0%) | 0,164  0,337  0,080  0,166  0,115  0,322  - | 40  20  15 | *“I had a hospital bed in our living room, so I could rest if that was needed. I did my shopping in the wheelchair, because the feeling of pressure on my vagina increased strongly when I walked distances longer than 500m, or stood for a long time.”*  “I laid down on the couch a few times a day, because only then the Braxton-Hicks contractions disappeared.”  *“Every time you see blood you are shocked, you should not have this in a normal pregnancy.”*  “*Every time I went to the toilet, I wiped with fear”.*  “My pelvic floor therapist said: after what you have been through, I am not surprised if you kept your pelvic floor so tight for such a long time, which is still bothering you.” |
| **ACTIVITIES AND PARTICIPATION** | | | | | | | | |
| **Chapter 4: mobility** | | | | | | | | |
| D410 | Changing basic body positions | Lying down (d4100)  - Bedrest  Difficulties finding balance in activities | 11 (100%)  6 (55%) | 6 (75%)  2 (25%) | 17 (89%)  8 (42%) | 0,164  0,208 | 40 | *“You just do not do anything, so afraid to do something that will make the pregnancy go wrong, because of your fault.”*  *“It is very difficult to find a balance in activity, the information and advice is very unclear about what is accepted to undertake in activities. This anxiety (doing/moving too much) is more intense with a little one walking around, because I cannot always take it easy.”* |
| D415 | Maintaining a body position | Maintaining a standing position (d4154) | 2 (18%) | 3 (38%) | 5 (26%) | 0,336 |  | *“I had troubles standing for a long time.”* |
| D430 | Lifting and carrying objects | Lifting (d4200) | 2 (18%) | 3 (38%) | 5 (26%) | 0,336 |  | *“I was not allowed to lift heavy things, which is very complicated with a toddler walking around. She wants a lot of attention.”* |
| D450 | Walking | Walking short distances (d4500)  Pain while walking | 4 (36%)  1 (9%) | 4 (50%)  0 (0%) | 8 (42%) | 0,449  0,579 |  | *“It did not feel comfortable to walk with the increased Braxton-Hicks contractions all the time.”*  *“If I did a lot during the day, that bothered me when I sat down in the evening: I had difficulties getting up and walking short distances.”*  *“it really hurts when I walk, I can only walk short distances, you really feel that weight on your cervix.”* |
| **Chapter 6: domestic life** | | | | | | | | |
| D640 | Doing housework | Cleaning living area (d6402) | 3 (27%) | 3 (38%) | 6 (32%) | 0,506 |  |  |
| D660 | Assisting others | Assisting others with self-care (d6600)  Feeling of guilt towards family | 5 (45%)  4 (36%) | 3 (38%)  0 (0%) | 8 (42%)  4 (21%) | 0,551  0,085 |  |  |
| **Chapter 7: interpersonal interactions and relationships** | | | | | | | | |
| D710 | General interpersonal interactions | Basic interpersonal interactions (d710)  - Family life  - Dependence on family, partner and friends  Complex interpersonal interactions (d720)  - Need for contact with fellow cerclage-patients | 8 (73%)  9 (82%)  3 (27%) | 5 (63%)  3 (38%)  6 (75%) | 13 (68%)  11 (58%)  9 (48%) | 0,506  0,067  0,055 | 17  18  15 | *“After our first baby (with cerclage) we tried to have another baby very soon, we did not want our daughter to consciously experiencing this period with me having bed-rest.”*  *“It changes your relationship; you and your partner really live like brother and sister.”*  *“My husband does all the physically demanding things, he takes care of the household and our other child, it is tough for him, but luckily we know that this is temporary.”*  *“Another cerclage-patient and I created a Facebook group together, where people can share experiences. They find support there, which I missed myself.”* |
| **Chapter 8: Major life areas** | | | | | | | | |
| D845 | Acquiring, keeping and terminating a job | Terminating a job (d8452)  - Partly or Completely | 10 (91%) | 8 (100%) | 18 (95%) | 0,579 | 22 | *“I stopped working during my pregnancy, my gynecologist said: go work half a day, otherwise the walls are closing in on you. But my job was not suitable and I did not trust myself in working half days, so it was easier to quit completely.”*  *“My life stopped for a number of years, with periods of doing everything in my reach to complete a successful pregnancy, to physical and mental recovery, to be able to work again for a while, and then this circle repeated. This probably killed my career.”*  *“After the second pregnancy loss, I got fired, because my employer said I had changed and no longer fit the team. That was tough.”*  *“I knew that I wanted to become pregnant again. Since it was going to be another high-risk pregnancy, I knew I would get back on sick leave. For that reason, it was hard to apply again.”* |
| **Chapter 9: Community, social and civic life** | | | | | | | | |
| D920 | Sports and leisure | Leisure (d9205)  - Limitation in social activities | 2 (18%) | 3 (38%) | 5 (26%) | 0,336 |  |  |
| **ENVIRONMENTAL FACTORS** | | | | | | | | |
| **Chapter 3: support and relationships** | | | | | | | | |
|  | Immediate family (e310)  Extended family (e315)  Friends (e320)  Health professionals (e355) | Feeling of incomprehension and misconnection with social environment  Lack of information provision  Lack of unambiguous information  Incomprehension of protocol  Communication between academic and regional hospital | 55 (45%)  6 (55%)  3 (27%)  5 (45%)  1 (13%) | 4 (50%)  8 (100%)  3 (38%)  2 (25%)  5 (63%) | 9 (47%)  14 (74%)  6 (32%)  7 (37%)  6 (32%) | 0,605  0,040  0,506  0,494  0,024 | 23  25  11  13  11 | *“People said: what a gift that you will deliver with a caesarean section. For me it was the only possibility to give birth to a child (abdominal cerclage), but I would rather have a vaginal delivery. It is not such a gift.”*  *“I find it difficult to talk about it, I notice that others find it difficult too. When I go to the supermarket, I notice people taking another path, so they don’t have to cross me.”*  *“My social environment dares or does not want to talk about it, they find it difficult to start the conversation or mention the name of my deceased son. That affects me; it feels like everyone has already forgotten him.”*  *“People in the social circle avoid talking to you because they don't know what a cerclage is, and if you explain it, people think and say: now that you have that cerclage stitch, it is fine right? But that is not true. You are also afraid with that stitch”*  *“Information provision could be much better: I really missed written information on all aspects of preterm birth: causes, symptoms, treatment options etcetera.”*  *“It would be nice if we all had the mailing address of a doctor specialized in cerclages, so that we can always contact this person with questions and several uncertainties.”*  *“I had agreed to myself not to cry at appointments, but after 15 weeks I really had to cry. My gynecologist asked me: do you feel safer if you are admitted to the hospital? I absolutely did not want to, but by saying this, I felt I was taken seriously”* |

The ICF-DH provides a structured insight into functioning and limitations, encompassing three key components: body functions and structures, activities and participation, and environmental and personal factors (see to figure 2). The coded data "B152, B160, etc." is a representation of the ICF codes linked to specific 'meaningful concepts' derived during the coding process.

**Appendix 6: Supplement Table 1, Questions of the evaluation form**

| 1. How did you experience participating in the group interview? 2. How did you experience sharing your own experiences in the group interview? 3. How did you feel about hearing the experiences of other patients in the group interview? 4. To what extend did you have recognition in the experiences of other patients on a scale from 0-10. Zero means no recognition and ten means a maximum recognition. 5. Did you experience support from participating in the group interview? 6. Did the group interview help you in your own processing? 7. How did you experience the atmosphere within the group interview? Did you have the feeling of confidentiality? 8. How did you experience the set-up and structure of the group interview? 9. Did you miss any relevant (substantive) topics during the focus group interview? 10. What went well and what are points of improvement for future focus group studies? 11. Do you have any other comments or suggestions you would like to share regarding the group interviews? |
| --- |
